# Downregulation of RPL15 may predict poor survival and associate with tumor progression in pancreatic ductal adenocarcinoma

**DOI:** 10.18632/oncotarget.5939

**Published:** 2015-10-15

**Authors:** Ting-Ting Yan, Xue-Liang Fu, Jiao Li, Ying-Nan Bian, De-Jun Liu, Rong Hua, Lin-Lin Ren, Cheng-Tao Li, Yong-Wei Sun, Hao-Yan Chen, Jing-Yuan Fang, Jie Hong

**Affiliations:** ^1^ Division of Gastroenterology and Hepatology, Renji Hospital, Shanghai Institution of Digestive Disease, Key Laboratory of Gastroenterology and Hepatology, Ministry of Health, State Key Laboratory of Oncogene and Related Genes, Shanghai Jiao-Tong University School of Medicine, Shanghai, China; ^2^ Department of Biliary-Pancreatic Surgery, Renji Hospital, Shanghai Jiao-Tong University School of Medicine, Shanghai, China; ^3^ Shanghai Key Laboratory of Forensic Medicine, Institute of Forensic Sciences, Ministry of Justice, Shanghai, China

**Keywords:** pancreatic cancer, RPL15, metastasis, biomarker, PDAC, Pathology Section

## Abstract

Early diagnosis and treatment in pancreatic ductal adenocarcinoma (PDAC) is still a challenge worldwide. The poor survival of PDAC patients mainly due to early metastasis when first diagnosed and lack of prognostic biomarker. Ribosomal protein L15 (RPL15), an RNA-binding protein, is a component of ribosomal 60S subunit. It was reported that RPL15 is dysregulated in various type of cancers. However, little is known about the role of RPL15 in PDAC carcinogenesis and progression. Herein, we clarified RPL15 expression status may serve as an independent prognostic biomarker in three independent PDAC patient cohorts. We found that RPL15 was dramatically decreased in PDAC tissues and cell lines. The high expression of RPL15 was inversely correlated with TNM stage, histological differentiation, T classification and vascular invasion. Low expression of RPL15 was significantly associated with poor overall survival of PDAC patients. Furthermore, we demonstrated that the reduction of RPL15 may promote invasion ability of pancreatic cell by inducing EMT process. In conclusion, decreased RPL15 expression is associated with invasiveness of PDAC cells, and RPL15 expression status may serve as a reliable prognostic biomarker in PDAC patients.

## INTRODUCTION

Pancreatic cancer is one of the most malignant and lethal cancers with increasing incidence and mortality worldwide [1]. It is the fourth and sixth leading cause of cancer-related deaths in USA and China, respectively [2, 3]. Among all pancreatic cancer cases, pancreatic ductal adenocarcinoma (PDAC) accounts for approximately 85%. Although a mass of efforts was made to improve the diagnosis and treatment of PDAC, it is still a refractory disease with 5-year survival rate approximately 5% and a median survival time (MST) of 6 months [4]. The poor prognosis of PDAC has been ascribed to early vascular dissemination and metastasis because of difficulty in early detection and insensitive to adjuvant therapy [5, 6]. Therefore, it is urgent to explore the potential mechanism to develop diagnostic and treatment strategy.

According to the emerging studies, approximately over 80 ribosomal proteins were found within the large and small subunits of eukaryotic ribosome [7]. Although the ribosomal proteins are supposed to mainly function in protein synthesis, recent studies have suggested that many ribosomal proteins yet demonstrate various extraribosomal functions in cell proliferation, DNA repair, apoptosis, drug resistance and other cellular processes [8-11]. As a component of the ribosomal 60S subunit, RPL15 was reported to be dysregulated in many types of diseases and cancers [12, 13]. Wang et al. found that overexpression of RPL15 participated in the carcinogenesis of esophageal cancer [14]. Additionally, it was reported that upregulation of RPL15 is associated with cell proliferation in gastric cancer [15]. However, another study revealed that compared to normal skin tissues, RPL15 was down-regulated in cutaneous squamous cell carcinoma, which was contrast to the former two studies [16]. To date, little is known about the role of RPL15 in PDAC. To determine the expression pattern of RPL15 in PDAC, we profiled the expression status of RPL15 in PDAC and corresponding noncancerous tissues. We further analyzed the association between RPL15 expression and clinicopathological characteristics in PDAC, and examined the role of RPL15 in PDAC invasion.

## RESULTS

### The expression of RPL15 is significantly downregulated in PDAC

**Figure 1:**
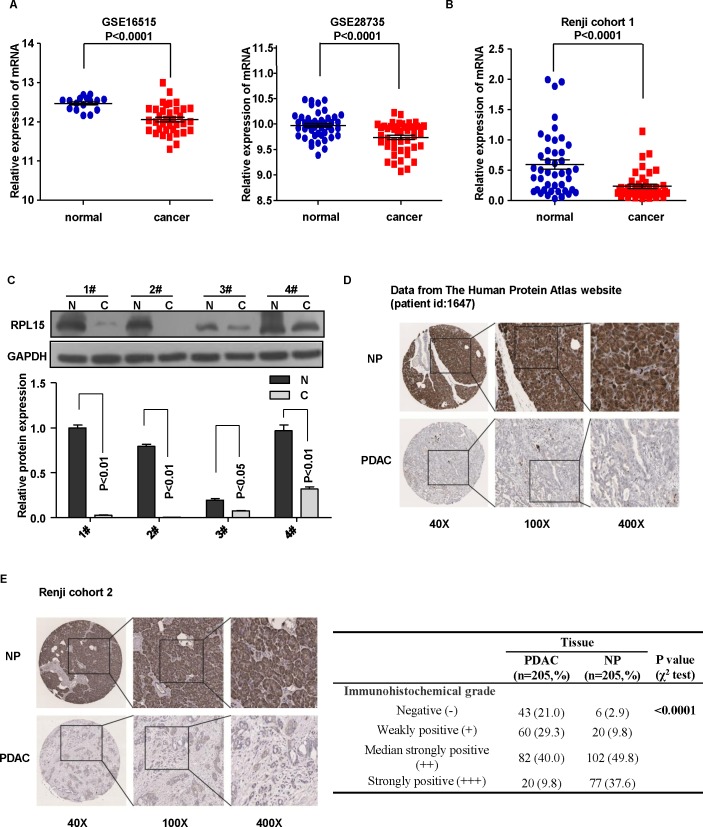
The expression of RPL15 in PDAC tissues **A.**-**B.** Detection of RPL15 expression in dataset GSE16515, GSE28735 and Renji cohort 1 patients. Error bars in the scatter plots represent SE. **C.** Western bolts showed RPL15 protein level in four paired tissue of patients from Renji cohort 1. **D.**-**E.** RPL15 protein level was measured by immunohistochemical analysis in normal pancreas and PDAC tissue from The Human Protein Atlas website and Renji cohort 2. RPL15: ribosomal protein L15; GAPDH: glyceraldehyde-3-phosphate dehydrogenase; NP: normal pancreas; PDAC: pancreatic ductal adenocarcinoma.

To evaluate the expression status of RPL15 in human PDAC tissues, we first analyzed two independent microarray datasets from GEO database [17-19]. The results demonstrated that the expression level of RPL15 was significantly reduced in tumor tissues compared with normal pancreatic epithelium in both independent datasets (*p* < 0.0001, Figure 1A). Then we analyzed the expression level of RPL15 in two independent patient cohorts from Renji Hospital. We first performed real-time PCR to measure the mRNA expression of RPL15 in PDAC tissues and paired adjacent normal tissues (*n* = 53, cohort 1, fresh tissues). Compared with adjacent normal tissues, PDAC tissues displayed lower mRNA expression level of RPL15 (*P* < 0.0001, Figure 1B). Furthermore, we detected the protein expression of RPL15 in four fresh pairs of PDAC tissues and adjacent normal tissues from cohort 1. Western blot data showed that RPL15 protein was obviously decreased in PDAC tissues (Figure 1C). Besides, data from The Human Protein Atlas website also demonstrated that RPL15 protein was not detected in most PDAC compared with normal pancreas (patient ID:1647, male, age 63, Figure 1D). We next measured RPL15 expression in 205 pairs of clinical PDAC samples by immunohistochemistry (*n* = 205, cohort 2, paraffin-embedded tissues). The tissue microarray analysis results also confirmed that RPL15 protein displayed lower expression in tumor tissues than peritumoral tissues (*p* < 0.0001, Figure 1E). These results indicated that both the mRNA and protein level of RPL15 were significantly downregulated in PDAC tissues.

### Associations of RPL15 expression with clinicopathological features and prognosis in PDAC patients

In order to determine the clinical significance of RPL15, we analyzed the relationship between RPL15 expression and clinicopathological characteristics of PDAC in Renji cohort 1 and cohort 2. As summarized in Table 1, RPL15 mRNA expression was significantly associated with histological differentiation (well/moderate *vs*. poor; *p* = 0.031) and vascular invasion (absent *vs*. present; *p* = 0.023) in Renji cohort 1 patients. Furthermore, the protein level of RPL15 was prominently correlated with TNM stage (I *vs*. II *vs*. III *vs*. IV, *p* = 0.033), T classification (T1-2 *vs*. T3-4; *p* = 0.035), vascular invasion (absent *vs*. present; *p* = 0.045) and histological differentiation (well *vs*. moderate/poor; *p* = 0.005) among Renji cohort 2 patients (Table 2); whereas no significant relevance was found with age, gender, tumor location, tumor size, CA199 level, lymph node metastasis and distant metastasis. It illustrated that, RPL15 expression was significantly correlated with carcinogenesis and invasion of PDAC.

**Table 1:** Correlations between RPL15 expression and clinicopathologic features in Renji cohort 1 patients with pancreatic ductal adenocarcinoma (PDAC)

		Expression of RPL15		
Clinicopathological feature	Total 53	Low (n=27, 50.2%)	High (n=26, 49.8%)	P value (χ^2^ test or Fisher's exact test)
**Age(years)**				
<65	26	10 (38.5)	16 (61.5)	0.074
≥65	27	17 (63.0)	10 (37.0)	
**Gender**				
Male	32	16 (50.0)	16 (50.0)	0.865
Female	21	11 (52.4)	10 (47.6)	
**Tumor location**				
Head	38	18 (47.4)	20 (52.6)	0.407
Body/tail	15	9 (60.0)	6 (40.0)	
**TNM stage(AJCC)**				
Stage I	13	6 (46.2)	7 (53.8)	0.378
Stage II	33	16 (48.5)	17 (51.5)	
Stage III	4	2 (50.0)	2 (50.0)	
Stage IV	3	3 (100.0)	0 (0.0)	
**Size**				
≤2cm	9	6 (66.7)	3 (33.3)	0.300
>2cm	44	21 (47.7)	23 (52.3)	
**T classificattion**				
T1,2	13	6 (46.2)	7 (53.8)	0.691
T3,4	40	21 (52.5)	19 (47.5)	
**Lymph node metastasis**				
Absent	34	16 (47.1)	18 (52.9)	0.449
Present	19	11 (57.9)	8 (42.1)	
**Distant metastasis**				
Absent	50	24 (48.0)	26 (52.0)	0.236
Present	3	3 (100.0)	0 (0.0)	
**Vascular invasion**				
Absent	47	21 (44.7)	26 (55.3)	**0.023**
Present	6	6 (100.0)	0 (0.0)	
**Histological differentiation**				
Well/Moderate	33	13 (39.4)	20 (60.6)	**0.031**
Poor	20	14 (70.0)	6 (30.0)	
**CA199 level(U/ml)**				
≤35	6	4 (66.7)	2 (33.3)	0.158
>35	36	13 (36.1)	23 (63.9)	
Missing	11			

Values in parentheses indicate percentage values. The bold number represents the *P*-values with significant differences.

**Table 2:** Correlations between RPL15 expression and clinicopathologic features in Renji cohort 2 patients with pancreatic ductal adenocarcinoma (PDAC)

		Expression of RPL15		
Clinicopathological feature	Total 205	Low (n=103, 50.2%)	High (n=102, 49.8%)	P value (χ^2^ test)
**Age(years)**				
<65	97	48 (49.5)	49 (50.5)	0.837
≥65	108	55 (50.9)	53 (49.1)	
**Gender**				
Male	117	60 (51.3)	57 (48.7)	0.732
Female	88	43 (48.9)	45 (51.1)	
**Tumor location**				
Head	139	71 (51.1)	68 (48.9)	0.729
Body/tail	66	32 (48.5)	34 (51.5)	
**TNM stage(AJCC)**				
Stage I	38	11 (28.9)	27 (71.1)	**0.033**
Stage II	132	71 (53.8)	61 (46.2)	
Stage III	21	13 (61.9)	8 (38.1)	
Stage IV	14	7 (50.0)	7 (50.0)	
**Tumor size**				
≤2cm	27	12 (44.4)	15(55.6)	0.518
>2cm	178	91 (51.1)	87 (48.9)	
**T classificattion**				
T1,2	42	15(35.7)	27 (64.3)	**0.035**
T3,4	163	88 (54.0)	75(46.0)	
**Lymph node metastasis**				
Absent	136	63 (46.3)	73 (53.7)	0.115
Present	69	40 (58.0)	29 (42.0)	
**Distant metastasis**				
Absent	191	96 (50.3)	95 (49.7)	0.985
Present	14	7 (50.0)	7 (50.0)	
**Vascular invasion**				
Absent	177	84 (47.5)	93 (52.5)	**0.045**
Present	28	19 (67.9)	9 (32.1)	
**Histological differentiation**				
Well	11	1 (9.1)	10 (90.9)	**0.005**
Moderate/poor	194	102 (52.6)	92 (47.4)	
**CA199 level(U/ml)**				
≤35	26	11 (9.1)	15 (90.9)	0.627
>35	105	50 (52.6)	55 (47.4)	
Missing	74			

Values in parentheses indicate percentage values. The bold number represents the *P*-values with significant differences.

**Figure 2:**
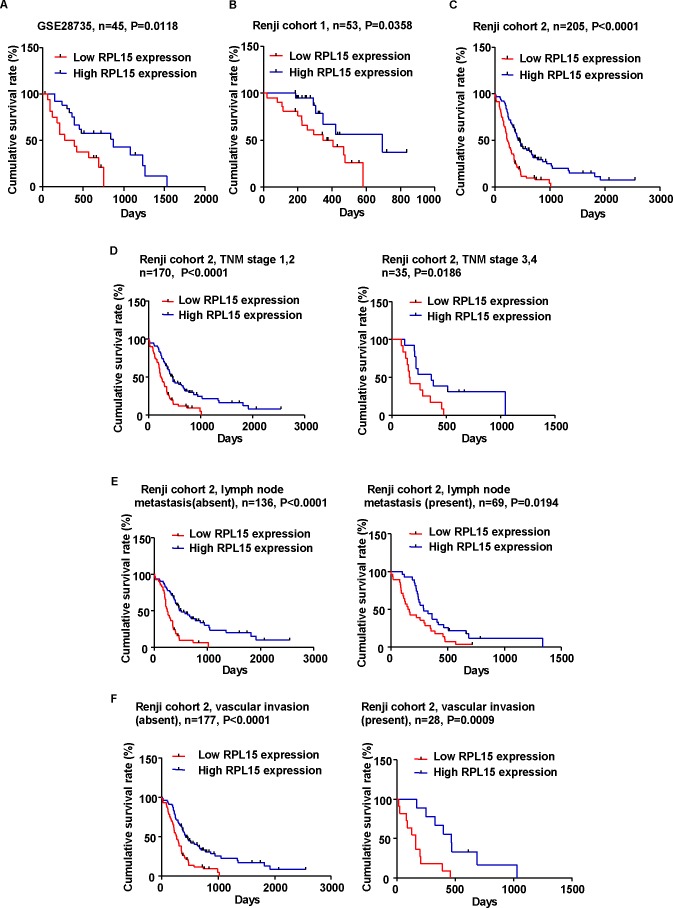
Kaplan-Meier analysis of overall survival in PDAC patients **A.**-**C.** The association of RPL15 expression and patient survival was conducted in GSE28735 dataset, Renji cohort 1 and Renji cohort 2. **D.**-**F.** Correlation between RPL15 expression and patient survival in Renji cohort 2 is independent of TNM stage, lymph node metastasis and vascular invasion.

To further define the role of RPL15 in PDAC prognosis, we measured the correlation of RPL15 expression with overall survival using Kaplan-Meier analysis and log-rank test in GSE28735, Renji cohort 1 and 2 patients, respectively. The results showed that RPL15 expression was dramatically associated with PDAC patients' overall survival in GSE28735 (*p* = 0.0118, Figure 2A), Renji cohort 1 (*p* = 0.0358, Figure 2B) and cohort 2 (*p* < 0.001, Figure 2C), which indicates that the overall survival is better in PDAC patients with high RPL15 expression than in those with low RPL15 expression. Besides, we also assessed the correlation between RPL15 expression and overall survival in different sub-groups according to TNM stage, status of lymphatic metastasis and vascular invasion in Renji cohort 2 patients. It turned out that the overall survival is shorter in PDAC patients with decreased RPL15 expression, regardless of TNM stage, lymphatic metastasis and vascular invasion (Figure 2D-2F).

**Figure 3:**
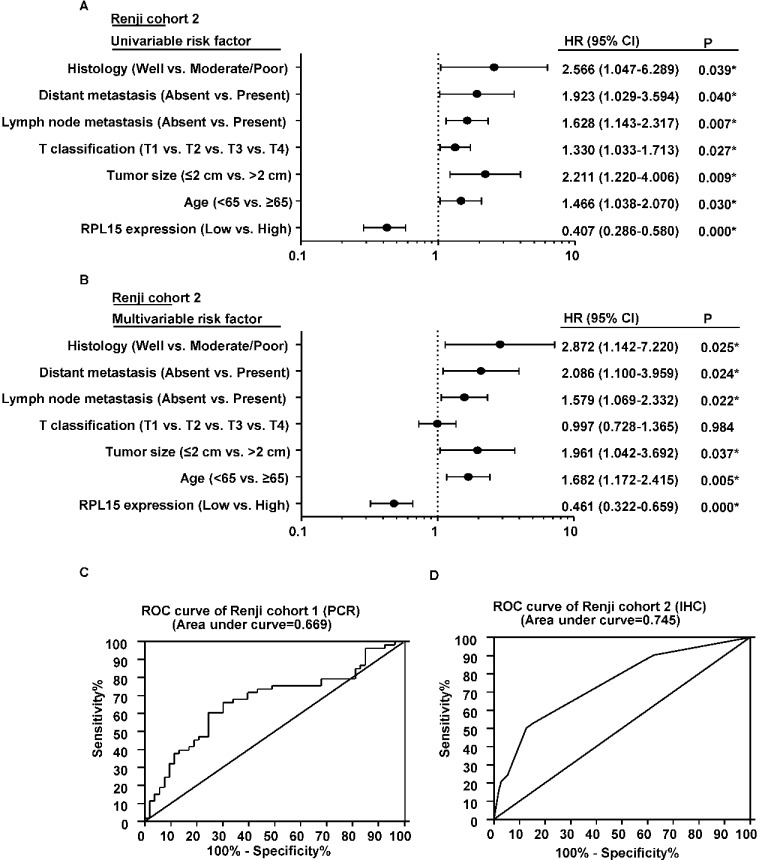
The potential value of RPL15 expression in predicting PDAC and patient prognosis **A.**-**B.** The forest plot showed the correlation between pancreatic patient overall survival and RPL15 expression as well as other clinical characteristics by using univariable and multivariable analysis. **C.**-**D.** ROC curve analysis from Renji cohort 1 and Renji cohort 2 according to RPL15 expression.

To directly identify the risk factors associated with patient overall survival, RPL15 expression level as well as 10 clinicopathological factors were evaluated by conducting Cox univariable and multivariable analyses in Renji cohort 2. As a result, the univariable analysis indicated that age (< 65 *vs*. ≥65), tumor size (≤2 cm *vs*. > 2 cm), T classification (T1 *vs*. T2 *vs*. T3 *vs*. T4), lymph node metastasis (absent *vs*. present), distant metastasis (absent *vs*. present), histological differentiation (well *vs*. moderate/poor) and expression of RPL15 (low *vs*. high) were significant prognostic factors for overall survival prediction (Table 3, Figure 3A). Moreover, the multivariable Cox regression analysis also confirmed that RPL15 expression, age, tumor size, lymphatic metastasis, distant metastasis and histological differentiation were independent predictors of PDAC patients' overall survival after pancreatectomy (Table 3, Figure 3B). Furthermore, receiver operating characteristic (ROC) curve analyses were conducted to investigate the prediction value of RPL15 expression. Results demonstrated that RPL15 was a practical predictor, with an area under curve (AUC) of 0.669 and 0.745 in Renji cohort 1 and Renji cohort 2, respectively (Figure 3C-3D). These data indicated that low RPL15 expression may be a predictor for diagnosis and prognosis in PDAC patients.

**Table 3:** Univariable and multivariable analyses of prognostic parameters for survival in Renji cohort 2 patients with pancreatic ductal adenocarcinoma (PDAC)

	Univariate analysis			Multivariate analysis		
Prognostic parameter	HR	95% CI	*P* value	HR	95% CI	P value
**Expression of RPL15** (low vs. high)	0.407	0.286-0.580	**0.000**	0.461	0.322-0.659	**0.000**
**Age** (<65 vs. ≥65)	1.466	1.038-2.070	**0.030**	1.682	1.172-2.415	**0.005**
**Gender** (male vs. female)	0.757	0.531-1.079	0.124	-	-	-
**Tumor Size** (≤2 cm vs. >2 cm)	2.211	1.220-4.006	**0.009**	1.961	1.042-3.692	**0.037**
**T classification** (T1-2 vs. T3-4)	1.330	1.033-1.713	**0.027**	0.997	0.728-1.365	0.984
**Lymph node metastasis** (absent vs. present)	1.628	1.143-2.317	**0.007**	1.579	1.069-2.332	**0.022**
**Distant metastasis** (absent vs. present)	1.923	1.029-3.594	**0.040**	2.086	1.100-3.959	**0.024**
**Vascular invasion** (absent vs. present)	1.554	0.954-2.532	0.077	-	-	-
**Tumor location** (head vs. body/tail)	0.983	0.683-1.416	0.928	-	-	-
**Histology** (well vs. moderate/poor)	2.566	1.047-6.289	**0.039**	2.872	1.142-7.220	**0.025**
**CA199 level** (≤35 U/ml vs. >35 U/ml)	1.411	0.817-2.438	0.217	-	-	-

HR: Hazard ratio; CI: Confidence interval. The bold number represents the *P*-values with significant differences.

### RPL15 decreases the invasion and metastasis ability of pancreatic cancer cell

**Figure 4:**
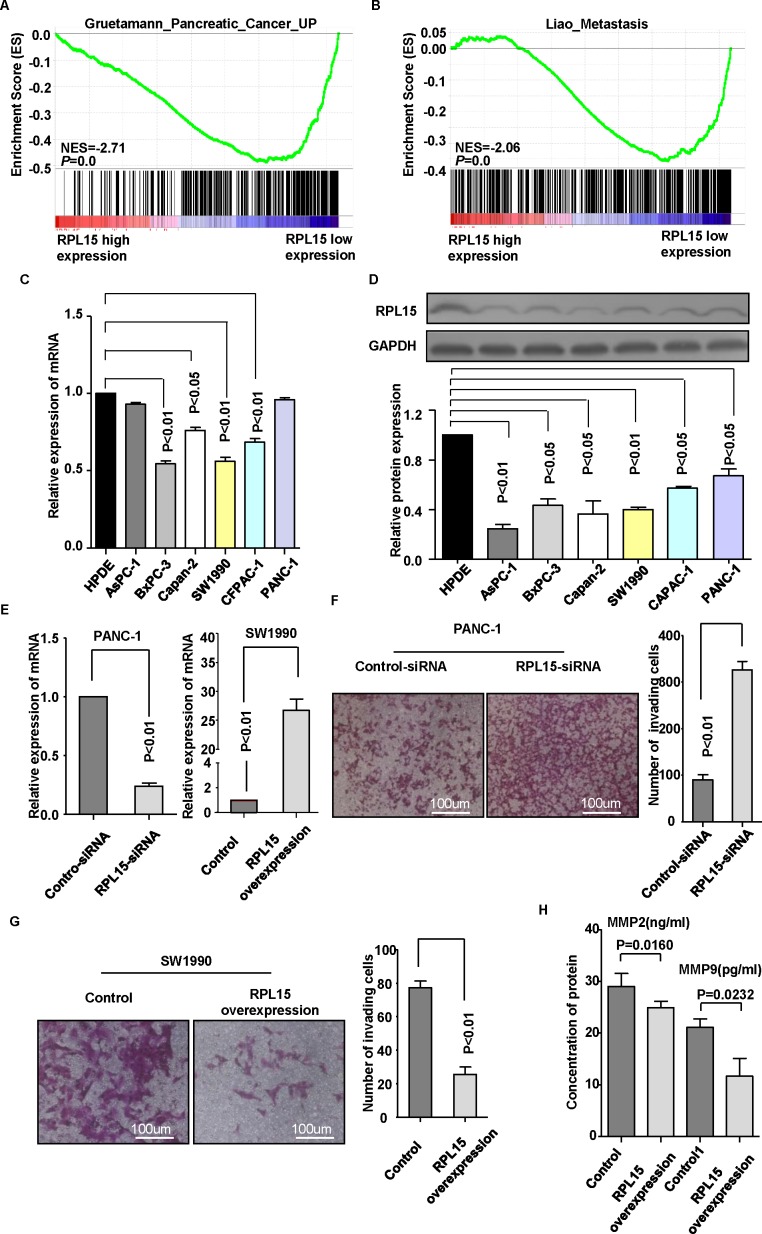
Overexpression of RPL15 inhibited pancreatic cancer cell invasion **A.**-**B.** GSEA in dataset GSE28735 revealed that “Gruetamann_Pancreatic_Cancer_UP” and “Liao_Metastasis” pathway were enriched in RPL15-low expression group compared with RPL15-high expression group. The enrichment score (ES, green line) means the degree to which the gene set is overrepresented at the top or bottom of the ranked list of genes. **C.**-**D.** RPL15 expression was measured in normal pancreatic cell line HPDE and six PDAC cell lines by real-time PCR and western bolt. **E.** Knockdown and overexpression efficiency was confirmed by real-time PCR in PANC-1 and SW1990 cell line, respectively. **F.**-**G.** Invasion ability was measured by transwell chamber assay in PANC-1 cell and SW1990 cell; Results were quantitated by counting invasive cells in four randomly selected high-power fields for three replicates. Scale bar =100um **H.** Influence of RPL15 overexpression on MMP2 and MMP9 secretion in SW1990 cells were evaluated by ELISA. Results shown are the mean±SD of triplicate determination from three independent experiments.

To gain insight into the biological pathways involved in pancreatic cancer pathogenesis stratified by the median of RPL15 expression, Gene Set Enrichment Analysis (GSEA) performed in GSE28735 dataset showed that “Gruetamann_Pancreatic_Cancer_UP” and “Liao_Metastasis” pathway were enriched in patients with RPL15-lower expression *versus* patients with RPL15-higher expression (Figure 4A-4B). To further clarify the biological functions of RPL15, cell proliferation assay and transwell chamber assay were performed to evaluate whether RPL15 may affect the cell proliferation and invasion in pancreatic cancer cells. First, we detected RPL15 expression in six pancreatic cancer cell lines and one normal pancreatic cell line both at mRNA and protein level. Results showed that RPL15 was significantly decreased in the pancreatic cancer cell lines, especially in cell line SW1990 (Figure 4C-4D). Therefore, we transfected SW1990 with RPL15 overexpression plasmids and PANC-1 with RPL15-siRNAs, respectively. The transfection efficiency was confirmed by real-time PCR (Figure 4E). The cell proliferation assay showed no difference between RPL15 overexpression and negative control (Supplementary Figure 1). Transwell invasion assay demonstrated that RPL15 overexpression notably blocked the invasiveness of SW1990, while RPL15-depleted PANC-1 cells had a prominently higher invasive capacity than negative control (Figure 4F-4G). These data revealed that overexpression of RPL15 could impair the invasion ability in pancreatic cancer cells, however it had no effect on cell proliferation of pancreatic cancer. To further verify RPL15′s role in PDAC metastasis, we established a PDAC metastasis model in nude mice. The SW1990-control stable cells and SW1990-RPL15 overxpression stable cells were subcutaneously injected through the right flank of nude mice, respectively. Hematoxylin-eosin (HE) staining showed that less metastatic PDAC foci were observed in the lungs of nude mice at 12 weeks after injection of SW1990-RPL15 overxpression stable cells, compared with SW1990-control stable cells (Supplementary Figure 2A-2B). In addition, we detected the MMP2 and MMP9 expression in cell supernatant and found that reductions in the expression of both MMP2 and MMP9 were observed in RPL15 overexpression cell compared with control-plasmid transfected cell (Figure 4H). Altogether, these results strongly support that RPL15 may play a critical role in PDAC cell invasion and metastasis.

### Effects of RPL15 overexpression on epithelial to mesenchymal transition process

**Figure 5:**
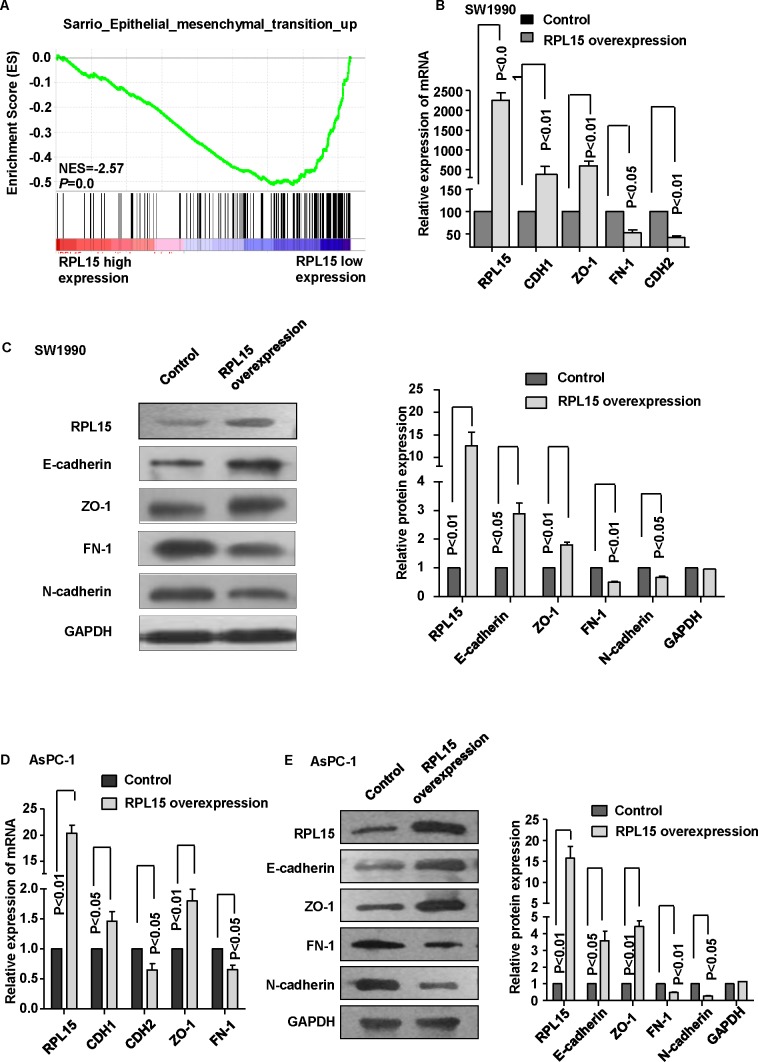
Overexpression of RPL15 reduces epithelial-mesenchymal transition (EMT) in pancreatic cancer cell line **A.** GSEA analysis in dataset GSE28735 revealed that EMT pathway was enriched in RPL15-low expression group compared with RPL15-high expression group. **B.** mRNA level of epithelial markers (CDH1 and ZO-1) and mesenchymal markers (CDH2 and FN-1) were measured by real-time PCR in SW1990 cell. **C.** Protein level of epithelial markers (E-cadherin and ZO-1) and mesenchymal markers (N-cadherin and FN-1) were measured by western blot in SW1990 cell. **D.** mRNA level of epithelial markers (CDH1 and ZO-1) and mesenchymal markers (CDH2 and FN-1) were measured by real-time PCR in AsPC-1 cell. **E.** Protein level of epithelial markers (E-cadherin and ZO-1) and mesenchymal markers (N-cadherin and FN-1) were measured by western blot in AsPC-1 cell. GAPDH was used as internal control. ZO-1: zonula occludens-1; FN-1: fibronectin-1; Results shown are the mean±SD of triplicate determination from three independent experiments.

Invasion and metastasis is a complex multistep process and epithelial-mesenchymal transition (EMT) is an important step during the development. To ascertain whether RPL15 regulate invasion *via* modulating EMT in PDAC, GSEA showed that the EMT-related gene signatures were more active in patients with lower RPL15 expression than in those with higher RPL15 expression (Figure 5A). Further real-time PCR and western blot data revealed that overexpression of RPL15 significantly increased CDH1 (E-cadherin) and ZO-1 expression, meanwhile reduced CDH2 (N-cadherin) and FN-1 expression in SW1990 and AsPC-1 cell (Figure 5B-5G). These data suggest a function role for RPL15 in repressing EMT, and this repression could result in the decrease of pancreatic cancer cell invasion and metastasis ability.

## DISCUSSION

Ribosomal protein family is a kind of the protein that, in conjunction with rRNA, make up the ribosomal subunits involved in the cellular process of translation and protein synthesis [20]. RPL15 belongs to the L15E family of ribosomal proteins and is a component of the 60S subunit [21]. Since RPL15 was first cloned and characterized from the crayfish orconectes limosus and petunia [22, 23], respectively, the biological roles of RPL15 have been investigated in several studies [15, 24, 25]. It has been reported that RPL15 may be dysregulated in various types of cancers, including esophageal, pulmonary and cutaneous carcinoma [14, 16, 26]. Here, we found that RPL15 may function as a tumor suppressor gene in PDAC progression since: 1) RPL15 expression is significantly decreased in PDAC tissues of multi-independent patients' datasets; 2) high RPL15 expression is positively correlated with better prognosis in three independent patients' cohorts; 3) GSEA data demonstrates that lower expression of RPL15 is more associated with PDAC pathogenesis.

Metastasis is a leading cause of cancer-related death. A majority of PDAC patients were found to have vascular invasion or distant metastasis when they were first diagnosed, resulting in unresection and poor survival. In our current study, we found that RPL15 may play an important role in PDAC invasion and metastasis. Firstly, we confirmed that metastasis genes were enriched in RPL15-low expression group compared with RPL15-high expression group by GSEA analysis. Besides, the invasion ability of SW1990 cells was remarkably attenuated due to overexpression of RPL15, while knockdown of RPL15 dramatically increased the number of invading cells in PANC-1 cell line. In addition, the *in vivo* experiment further confirmed that upregulation of RPL15 could significantly suppress lung metastasis process in nude mice. Moreover, the overexpression of RPL15 led to decreased level of MMP2 and MMP9 in SW1990 cell line. These data suggeste that decreased expression of RPL15 might involve in the invasion and metastasis of PDAC.

Besides, we determined the molecular mechanism by which overexpression of RPL15 inhibited pancreatic cancer cell invasion. Growing evidence has demonstrated that EMT plays a pivotal role in the process of cancer cell invasion and metastasis in various types of tumor, including PDAC [27-30]. Previous studies revealed that EMT process mediated the transition from immotile epithelial cells to migratory mesenchymal cells, which initiates and accelerates tumor invasion and metastasis [31, 32]. Beyond that, it could be characterized by the reduced epithelial markers and promoted mesenchymal markers. Here we showed that the overexpression of RPL15 could significantly upregulate epithelial markers and downregulate mesenchymal markers in pancreatic cancer cells. Hence, the impaired pancreatic cancer cell invasion ability due to overexpression of RPL15 could be accounted, at least in part, for the inhibition of EMT process. However, the direct regulation mechanism between RPL15 and EMT initiation needed to be more explored in our future study.

In a word, our results demonstrated that RPL15 was significantly downregulated in PDAC tissues and RPL15 expression could serve as an independent prognostic factor among PDAC patients. Furthermore, we found that overexpression of RPL15 could inhibit pancreatic cancer cell invasion and metastasis ability *via* suppressing EMT process. While the specific mechanism, which would be explained in our future research, remains to be elucidated. Therefore, our study revealed that RPL15 may have the potential to be a biomarker and could contribute to develop a novel effective strategy in PDAC diagnosis and therapy.

## MATERIALS AND METHODS

### Clinical specimen collection

A total of 53 freshly-frozen primary PDAC and matched adjacent non-tumor tissues (Cohort 1) were collected from patients who underwent pancreatic surgical resection at Renji Hospital (Shanghai, China) between January 2012 and December 2013. Tissue microarrays consisting of 205 PDAC specimens and corresponding noncancerous tissues (Cohort 2) were obtained from Renji Hospital from January 2002 to June 2013, and the pathological information was retrieved from the Pathology Department of Renji Hospital. All the patients were provided with written informed consent before enrolment, and the study was approved by the Research Ethics Committee of Shanghai Jiao Tong University School of Medicine Renji Hospital. None of the patients had received tumor-specific therapy before diagnosis. In the cohort 1 cases, 21 females and 32 males with age ranging from 38 to 91 were included. In the cohort 2 cases, there were 88 females and 117 males, aging from 38 to 90. The follow-up time was calculated from the date of surgery to PDAC-related death, or June 30, 2014, the ultimate deadline.

### GEO sample acquisition and GSEA analysis

The dataset of expression profiles and corresponding clinical information used in this study were downloaded from the Gene Expression Omnibus (GEO). Data extraction was performed with R 3.0.2 software.

To gain further insight into the biological pathways involved in PDAC progression through RPL15 pathway, a gene set enrichment analysis (GSEA) was performed. The canonical pathways gene sets (c2.cp.v4.0.symbols.gmt) from the Molecular Signatures Database-MsigDB (http://www.broad.mit.edu/gsea/msigdb/index.jsp) were used for enrichment analysis. Only gene sets represented by at least 15 genes were retained [33].

### Cell culture and treatment

Human pancreatic cancer cell lines SW1990, PANC-1, BxPC-3, AsPC-1, Capan-2, and CFPAC-1 were purchased from the Cell Resource Center, Shanghai Institute of Biochemistry and Cell Biology at the Chinese Academy of Sciences (Shanghai, China) and an immortalized normal human pancreatic ductal epithelial cell line HPDE6-C7 was obtained from Professor ZG Zhang (Shanghai Cancer Institute, Shanghai, China). Cells were maintained in complete growth medium recommended by the provider containing 10% fetal bovine serum (FBS), 100 U/ml penicillin and 100 μg/ml streptomycin and were incubated at 37°C in a 5% CO2 atmosphere.

The siRNAs targeting human RPL15 were transfected into the pancreatic cancer cells using the DharmaFECT 1 siRNA transfection reagent (Thermo Scientific Dharmacon lnc.), whereas nonspecific siRNA acted as negative controls. The FuGENE transfection reagent (Promega, Madison, WI, USA) was implemented in the RPL15 overexpressing plasmids transfections according to the manufacturer's protocol, while the empty plasmid was used as negative control. The siRNAs and the plasmids were purchased from Genepharm Technologies (Shanghai, China) and Genearray Biotechnology (Shanghai, China), respectively.

### Total RNA extraction and quantitative real-time PCR

Total RNA was extracted from tissues (Cohort 1) and cells by using Trizol reagent (Takara, Japan) according to the manufactuer's instruction. cDNA was synthesized using a PrimeScript RT Reagent Kit (Takara, Japan) in accordance with the protocol of manufacturer. StepOne Real-Time PCR System (Applied Biosystems, Grand Island, NY, USA) was applied to detect the expression level of target gene using the SYBR Premix Ex Taq II (Takara, Japan), and GAPDH acted as an internal control. The data was calculated by the 2^−ΔΔCt^ method [34]. Primer sequences are listed as follows.

**Table:** 

Gene	Forward primer	Reverse primer
RPL15	5′-CTGGGTTGGTGAAGATTCCA-3′	5′-GTGGACTGGTTTGGTGATCC-3′
E-cadherin	5′-AGAACGCATTGCCACATACACTC-3′	5′-CATTCTGATCGGTTACCGTGATC-3′
ZO-1	5′-CAACATACAGTGACGCTTCACA-3′	5′-GACGTTTCCCCACTCTGAAAA-3′
N-cadherin	5′-GGAGACATTGGGGACTTCATT-3′	5′-TCCTGCTCACCACCACTACTT-3′
Fibronectin	5′-GCCTTCAAGTTCCCCTGTTAC-3′	5′-GACTCTCTCCGCTTGGATTCT-3′
GAPDH	5′-GCATTGCCCTCAACGACCAC-3′	5′-CCACCACCCTGTTGCTGTAG-3′

### Immunohistochemistry

The tissue microarray sections were rehydrated and treated with 3% hydrogen peroxide, followed by antigen retrieval. After being blocked with 10% normal goat serum for 30 min, the sections were incubated with primary antibodies at 4°C overnight, followed by incubation with a peroxidase-labeled secondary antibody for 30 min at room temperature. Finally, Diaminobenzidine tetrahydrochloride (DAB; Maixin Biotech, China) was used for the color-reaction followed by nucleus counterstaining with hematoxylin. The following antibodies were used: rabbit anti-RPL15 (1:200, Abcam, UK); Elivision plus Polyer HRP (Mouse/Rabbit) IHC Kit (Maixin Biotech, China).

The slides were inspected independently by two investigators in a blinded manner. Protein expression was evaluated according to the extent and intensity of staining (percentage of positive cells was measured on a scale of 0-4: 0, 0-5%; 1, 5-25%; 2, 26-50%; 3, 51-75%; 4, 76-100%; the intensity of staining was measured on a scale of 0-3: 0, no staining; 1, weak staining; 2, moderate staining; 3, strong staining). And a final score was created to determine the cut-off value for low and high expression group by using grades of the extent × grades of intensity staining. Then the protein expression was sorted into four categories: “-” for a score of 0-3, “+” for a score of 3-6, “++” for a score of 6-9 and “+++” for a score of > 9; low expression was defined as a final score < 6 and high expression with a final score ≥ 6.

### ELISA

MMP2 and MMP9 level in the cell supernatants were detected quantitatively by using a human MMP2 and MMP9 ELISA kit (RayBiotech, USA) as described by the manufacturer.

### Western blot

Cells were lysed with RIPA (Beyotime, China) containing a protease inhibitor mixture (protease inhibitors; phosphatase inhibitors; PMSF; KangChen, Shanghai, China) on ice for 30 min. The concentration of protein was measured by a Bradford protein assay kit (Bio-Rad, Hercules, CA, USA) [35]. Proteins were separated on 10% SDS-polyacrylamide gels and transferred on to polyvinylidene difluoride (PVDF) membranes (Millipore, Bedford, MA, USA). Then the membranes were blocked with 5% nonfat milk and incubated with the primary antibodies at 4°C overnight. After that, the membranes were washed with TBST and incubated with HRP-conjugated goat anti-rabbit or goat anti-mouse IgG (1:3000, KangChen, Shanghai, China) for 1h. Finally, the ECL detection system (SuperSignal West Femto Maximum Sensitivity Substrate, Thermo Fisher Scientific, IL, USA) was used for visualization. Sources of antibodies and concentrations used were as follows: rabbit anti-RPL15 (1:1000, Abcam, UK), rabblt anti-E-cadherin (1:1000, CST, USA); rabbit anti-ZO-1 (1:1000, Abcam, UK); rabbit anti-N-cadherin (1:500, Abcam, UK); mouse anti-fibronection (1:1000, Abcam, UK), GAPDH (1:1000, KangChen, Shanghai, China).

### Cell proliferation assay

Cells were cultured in 96-well plates and treated with Cell Counting Kit-8 (CCK8, Dojindo Molecular Technologies, Kyushu, Japan) for 2 hours at the time points of 24 h, 48 h, and 72h after the initial planting. Cell absorbance was detected at 450nm with a microplate reader.

### Transwell chamber assay

After the chamber was coated with fresh Matrigel (diluted in 1:4 with serum-free medium) (BD Bioscience San Jose, CA, USA), 2 × 10^5^ cells suspended in serum-free medium were plated on the top of each chamber, while medium containing 20% FBS was put in the lower chamber, used as a chemo-attractant. After 48h incubation, cells that did not pass through the filter were removed by a cotton swab, whereas cells on the lower surface of the filter were fixed and stained with methanol and crystal violet, respectively. Finally, cells across through the membrane were counted using a microscope.

### The PDAC metastatic animal model

SW1990 cells were infected with lenti-RPL15 virus, lenti-control virus to construct the SW1990-RPL15 overexpression, SW1990-control stable cell lines, respectively. SW1990-control and SW1990-RPL15 overexpression cells were injected subcutaneously into the dorsal right flank of 4-week-old male BALB/c nude mice. After 12 weeks, Lungs of the nude mice were obtained post-mortem for HE staining. All experimental procedures were approved by the Institutional Animal Care and Use Committee.

### Statistical analysis

Statistical analysis was performed with the SPSS 13.0 software (SPSS Inc, Chicago, IL, USA). For clinicopathological analysis, the chi-squared test or Fisher's exact test were used. Survival curves were constructed by using the Kaplan-Meier method and analyzed by the log-rank test. Cox proportional hazards model was used to identify the prognostic factors by univariable and multivariable analysis. All the experiments were repeated at least three times. Results were considered to be significant if the P value was under 0.05.

## SUPPLEMENTARY MATERIAL FIGURES


