# Supplementary material for: Downregulation of RPL15 may predict poor survival and associate with tumor progression in pancreatic ductal adenocarcinoma
Source: Oncotarget. 2015 Oct 15;6(35):37028–42. doi: 10.18632/oncotarget.5939 (PMC4741913; doi:10.18632/oncotarget.5939)
Supplement: Supplementary file 1 [file oncotarget-06-37028-s001.pdf]

Downregulation of RPL15 may predict poor survival and associate with tumor progression in pancreatic ductal adenocarcinoma

Supplementary Material

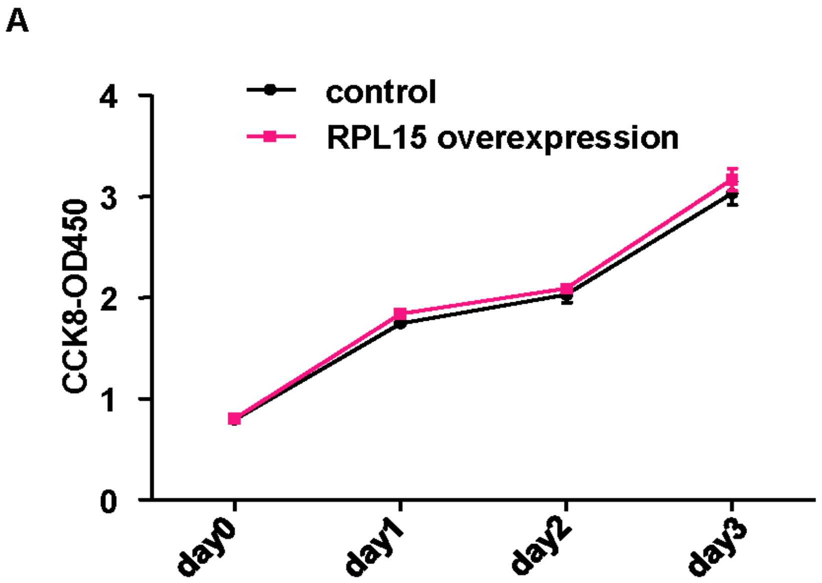

Figure S1: RPL15 overexpression had no effect on the proliferation of pancreatic cancer cell. (A) CCK8 assay of SW1990 cells transfected with RPL15 overexpression plasmids or control plasmids was performed. The absorbance was measured once a day.

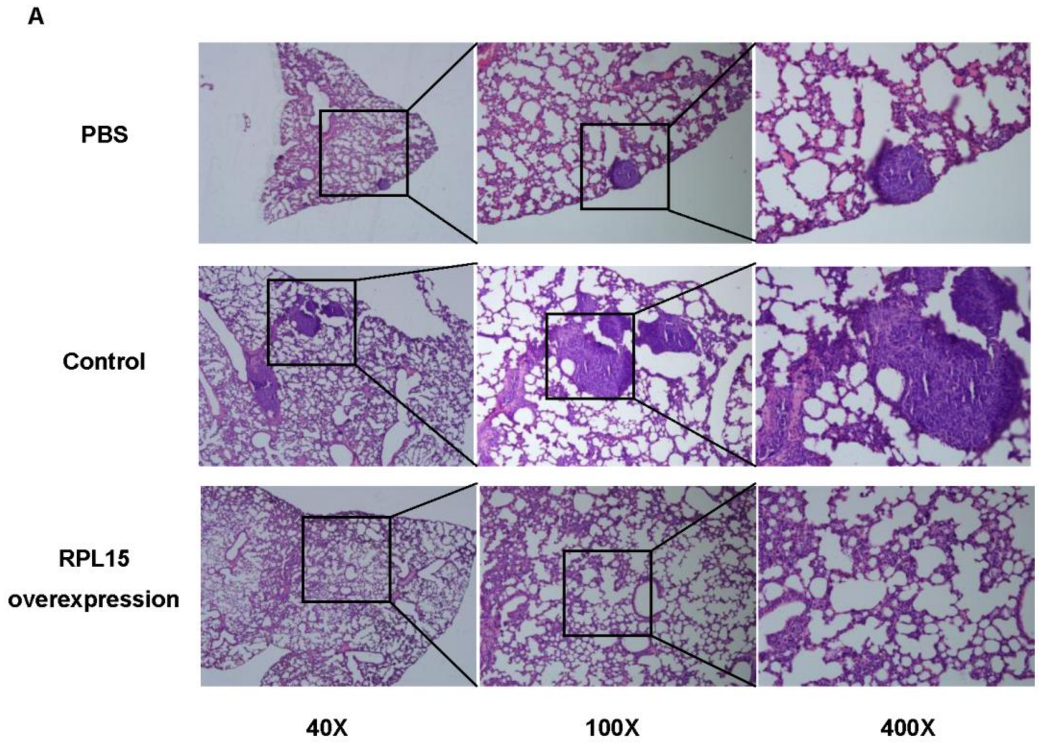

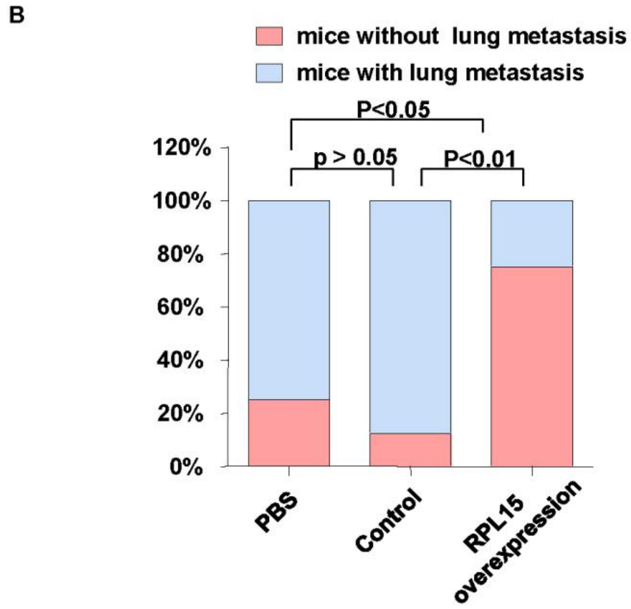

Figure S2: The funtional impact of RPL15 in vivo. (A) Representative HE images of lungs of nude mice in PBS group, control group and RPL15-overexpression group. (B) Fewer metastatic pancreatic cancer cells were detected in the lungs of nude mice at 12 weeks after injection of the SW1990-RPL15 group, compared with SW1990 control group. Control: SW1990-control virus group.
